# The impact of input node placement in the controllability of structural brain networks

**DOI:** 10.1038/s41598-024-57181-0

**Published:** 2024-03-22

**Authors:** Seyed Samie Alizadeh Darbandi, Alex Fornito, Abdorasoul Ghasemi

**Affiliations:** 1 Department of Computer Engineering, K. N. Toosi University of Technology, Tehran, Iran; 2 The Turner Institute for Brain and Mental Health, School of Psychological Sciences, and Monash Biomedical Imaging, Monash University, Clayton, Victoria Australia

**Keywords:** Complex systems, Brain networks, Structural controllability, Control energy, Computer science, Statistical physics, thermodynamics and nonlinear dynamics, Computer science, Statistical physics, thermodynamics and nonlinear dynamics

## Abstract

Network controllability refers to the ability to steer the state of a network towards a target state by driving certain nodes, known as input nodes. This concept can be applied to brain networks for studying brain function and its relation to the structure, which has numerous practical applications. Brain network controllability involves using external signals such as electrical stimulation to drive specific brain regions and navigate the neurophysiological activity level of the brain around the state space. Although controllability is mainly theoretical, the energy required for control is critical in real-world implementations. With a focus on the structural brain networks, this study explores the impact of white matter fiber architecture on the control energy in brain networks using the theory of how input node placement affects the LCC (the longest distance between inputs and other network nodes). Initially, we use a single input node as it is theoretically possible to control brain networks with just one input. We show that highly connected brain regions that lead to lower LCCs are more energy-efficient as a single input node. However, there may still be a need for a significant amount of control energy with one input, and achieving controllability with less energy could be of interest. We identify the minimum number of input nodes required to control brain networks with smaller LCCs, demonstrating that reducing the LCC can significantly decrease the control energy in brain networks. Our results show that relying solely on highly connected nodes is not effective in controlling brain networks with lower energy by using multiple inputs because of densely interconnected brain network hubs. Instead, a combination of low and high-degree nodes is necessary.

## Introduction

The brain is a complex system constructed from billions of neurons linked by complicated patterns of white matter fibers^1–3^. Cognitive function results from dynamical transitions of the neurophysiological activity in support of complex behaviours^4^. Recognizing the mechanisms and processes of these transitions by controlling the dynamics of brain networks is critical for many applications with medical and therapeutic purposes like discovering how the system can be altered by disease states, intervening in disease to redirect changes^5^, development^6^, and rehabilitation^7^.

We can consider a spatially contiguous ensemble of neurons as a region. We approximated the dynamics of cognitive functions through these regions with linear models that are useful to characterize how the anatomical structure of the brain influences its dynamic functions^8–10^. In this respect, a brain can be modeled by a graph. In brain networks, each brain region is represented by a network node, with each node having a state that signifies the neurophysiological activity level of that region at a specific time^11,12^. Furthermore, anatomical connections are represented by links between nodes. See Ref.^13^ for more details of modelling brain networks as graphs. Controllability refers to the ability to steer the state of the system, i.e., the state of all network nodes, from an initial state to a desired one in a finite time by driving certain nodes^14–16^. These specific nodes that need to be driven by external signals are called input nodes. Signals used in the controllability of brain networks may include electrical stimulation^17^ or task modulation^18^.

The first question in investigating brain controllability concerns how many and which nodes should receive external signals to control the network. The smallest required number of external signals to ensure controllability can be easily determined by solving a graph-based problem called maximum matching^14,19,20^. See Refs.^21–25^ for a more technical introduction and the relationship between controllability and structural features of networks. Brain networks need only a few input nodes to guarantee controllability^25,26^. In particular, brain networks are theoretically controllable by one region as an input node, and driving any brain region by a proper external signal can move the brain state from its initial state to the desired one.

However, determining the required input nodes to ensure controllability is just a theoretical approach, and it does not provide any information about the energy required for controlling the network in reality. In practice, the energy required for brain controllability following input to a single region may be unrealistic^26,27^. This is a critical consideration, given the high metabolic expense of brain functions. The required energy to control brain networks also varies across different input nodes^26^. Understanding the energetic cost of brain controllability is thus essential to developing plausible strategies for directing brain activity toward desired states, such as in brain therapies. Here, three questions arise: (i) what are the energy requirements of different input nodes to establish controllability, (ii) what is the reason for regional differences in the required energy, and (iii) how can we reduce the required control energy?

Studies have been conducted to analyze brain regions based on their ability to control brain networks^26^. One of the outcomes is that hubs, regions with many connections to other parts of the brain, have the ability to move the state of the brain into different states with little effort. However, the reason behind that has remained unclear. Some studies have investigated the effect of external signals and other parameters on the energy required^28–30^. It’s important to note that both the structural architecture of the brain and its functional networks are being studied, and control theory is being used to study both perspectives of brain networks. However, most frameworks used to study brain networks have overlooked the presence of neural noise, which is a crucial part of information processing^31^. This can lead to inaccurate control energy estimates. To address this issue, a recent study^32^ has revised the brain’s network control framework to incorporate stochasticity in linear systems. Moreover, studies have not considered the feedback mechanisms that regulate internal network control. A recent study has suggested a closed-loop perspective that relies on sparsity promoting optimal feedback control to explain the internal control mechanisms of the brain^33^. This perspective acknowledges that feedback information comes at a cost and suggests that a controller seeking to optimize processing efficiency must balance control performance and controller sparsity. Although research has revealed the capacity of different brain regions to control brain networks with varying amounts of energy under diverse circumstances, it does not explain the cause of this variation, nor does it suggest ways to decrease energy requirements. Furthermore, using only control theory methods to investigate the two remaining questions leads to NP-hard problems that are difficult to solve^34,35^. Hence, we need to employ other measures to investigate more about the energy in brain networks.

In this study, we show how the structure of white matter fibers affects the energy required to control brain networks by using the concept of the Longest Control Chain (LCC) in the network, i.e., the longest distance between input and non-input nodes. Numerically and theoretically, it’s shown that the control energy strongly correlates with the LCC in the network^36–38^. We also have indicated how input node placement can modify the LCC in the network to reduce the required energy of controllability, so the network structure and the location of inputs play a significant role in determining the control cost^35^. Here, we leverage this result to provide insight into the relationship between regional differences in energy needed to control structural brain networks and white matter fibers architecture. Additionally, we build on our prior work to suggest a solution to decrease the needed energy to control brain networks by applying additional external signals to the minimum number of regions to reduce the LCC in the network. Our work thus provides a formal bridge that links white matter architecture, controllable dynamics, and the energetics of brain function to study the difference in regional control energy and to reduce the cost of structural controllability of brain networks.

## Theoretical framework and data

### Network controllability and control energy

Network control theory offers a promising mathematical framework for linking the structure of a network to its dynamic behavior. To formally define the model, consider a directed network $\mathcal {G}(\mathcal {V}, \mathcal {E})$ where $\mathcal {V}=\{v_i | i=1,..., N\}$ is the set of nodes and $\mathcal {E} \subset \mathcal {V} \times \mathcal {V}$ is the set of weighted directed links, i.e., each link $(v_i\rightarrow v_j)$ has an associated weight $a_{ji}\in \mathbb R$ representing the strength of the interaction. We assign a state $x_i\in \mathbb R$ to each node $v_i$ which evolves following the equation

1 $$\begin{aligned} \dot{\textbf{x}}(t) = \textbf{A} \textbf{x}(t) + \textbf{B} \textbf{u}(t), \end{aligned}$$

where the first term on the right-hand side represents the internal dynamics of the system and the second term expresses the external control imposed on the network. Specifically, $\textbf{x}={[x_1, x_2, ..., x_N]}^T$ is the state of the system as a vector of all node states, $\textbf{A} \in \mathbb R^{N\times N}$ is the weighted adjacency matrix that shows the interaction between network nodes, $\textbf{u}(t)={[u_1, u_2, ..., u_M]}^T\in \mathbb R^M$ is a vector of *M* time-dependent control signals, and matrix $\textbf{B}\in \mathbb R^{N\times M}$ defines how the control signals are coupled to the system. In controllability of human brains, the state of the system $\textbf{x}(t)$ reflects the magnitude of the neurophysiological activity of the *N* brain regions at a specific time, control signals $\textbf{u}(t)$ correspond to different inputs that change the states of the system such as electrical stimulation or task modulation, the input matrix $\textbf{B}$ indicates brain regions whose states are modified by external signals, and adjacency matrix $\textbf{A}$ encodes the white matter connections between different brain regions as derived using methods detailed in the next section (“Data acquisition and brain network construction” section).

A system $(\textbf{A},\textbf{B})$ is controllable if, with the proper choice of control signals, we can drive it from any initial state $\textbf{x}(0)$ to any final state $\textbf{x}(t_\text {f})$ in finite time. Determining controllability, in general, is a numerically unstable problem that requires exact knowledge of the link weights, making it difficult to study real networks directly. Structural controllability provides a framework for examining controllability when we lack precise information about the strength of connections within a network. This may be due to errors in determining the strength or an inability to evaluate it. In such cases, structural controllability allows for the analysis of network controllability based on its structure, i.e., the presence or absence of connections, independent of communications weight^39^. The key argument of structural controllability is that if a network is structurally controllable, it is controllable for most weight assignments for connections^14,39^. In brain networks, the weight of connections between regions is either unknown or inferred with noisy estimates, making it hard to determine the exact strength of connections^40–42^. This can lead to inaccuracies in determining network controllability. To avoid this issue, we chose to examine the structural controllability of brain networks.

**Figure 1:**
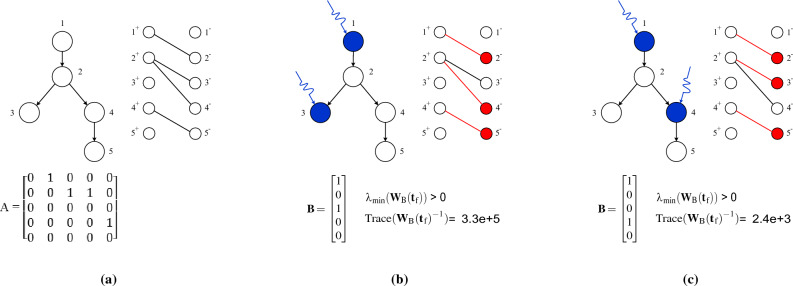
The structural controllability and the effect of LCC on the control energy. **(a)** the directed network $\mathcal {G}$, its bipartite graph $\mathcal {B}$, and the structural adjacency matrix $\textbf{A}$ of $\mathcal {G}$. **(b)** a controllability scheme in which the matching set $\mathcal{E}_\text {M} = \{(1^+,2^-),(2^+,4^-),(4^+,5^-)\}$ is shown using red links in the corresponding bipartite graph. The $\mathcal{E}_\text {M}$ is the maximum number of links for which no two links share the same start or end node. In this scheme, nodes 1 and 3 are unmatched nodes to apply independent external signals to control the network. The distance of network nodes from the closest input is [0, 1, 0, 2, 3], respectively, so the LCC equals three. **(c)** Since the maximum matching implementation is not unique, we selected another possible matching set in which nodes 1 and 4 are inputs. Note that the number of unmatched nodes in all possible maximum matching implementations is the same but with different unmatched nodes. In this case, the distance between inputs and network nodes is [0, 1, 2, 0, 1], respectively, so the LCC equals two. As is shown, the control scheme with a smaller LCC needs less control energy than the one with a larger LCC.

To investigate the structural controllability of a network, we require the structural adjacency matrix, which is a binary matrix indicating whether there is a connection between nodes *i* and *j* based on whether $a_{ij}=1$ or $a_{ij}=0$^14,39^. We use the weighted adjacency matrix obtained by DTI to find the connections and convert any $a_{ij}>0$ to 1 to obtain the structural adjacency matrix $\textbf{A}$ of brain networks. Results in control theory show that network $\textbf{A}$ given a set of input nodes is structurally controllable if the smallest eigenvalue of the controllability Gramian matrix $\lambda _\text {min}(\textbf{W}_\text {B}(t_\text {f}))$ is larger than zero where ${\textbf{W}_\text {B}}(t_\text {f})=\int _{0}^{t_\text {f}} e^ {\textbf{A} \tau } \textbf{B} \textbf{B}^T e^ {\textbf{A}^T\tau }d\tau$  ^43–45^. Finding the minimum required input nodes to ensure structural controllability is an NP-complete problem^46^, however, if a control signal can couple to multiple nodes, the minimum number of external signals to ensure structural controllability can be determined in polynomial time by the maximum matching algorithm in the bipartite graph of $\mathcal {G}$. The bipartite representation $\mathcal {B}$ of a directed network $\mathcal {G}$ can be shown by splitting each node $v\in \mathcal {V}$ into two copies $v^+\in \mathcal {V}^+$ and $v^-\in \mathcal {V}^-$. If there exists a directed link $(v\rightarrow w)$ in $\mathcal {G}$, we add an undirected link $(v^+ - w^-)$ to $\mathcal {B}$. A matching in the bipartite graph $\mathcal {B} (\mathcal {V}^+ \cup \mathcal {V}^-, \mathcal{E}')$ is a subset of links $\mathcal{E}_\text {M} \subset \mathcal{E}'$ such that no two links in $\mathcal{E}_\text {M}$ share start or endpoints, and a node in the set $\mathcal {V}^-$ is unmatched if no link in $\mathcal{E}_\text {M}$ is pointing at it. See Fig. 1 for an illustrative example. Therefore, if $N_\text {i}$ shows the number of external signals and $N_\text {u}$ indicates the number of unmatched nodes in the maximum matching, then

2 $$\begin{aligned} N_\text {i} = max (1,N_\text {u}). \end{aligned}$$

Solving the maximum matching problem provides a set of nodes to control the system; however, it does not provide any information about the required energy for controllability. The energy of controllability for a given set of input nodes can be measured using various metrics. The average energy and the worst-case energy, based on the controllability Gramian matrix, are the most commonly used metrics. If we want to determine the average energy required to steer the network towards all possible final states, we can analyze the Trace of the inverse of the controllability Gramian matrix, denoted as $\text {Trace}({\textbf{W}_\text {B}(t_\text {f})}^{-1})$^47^. On the other hand, for the worst-case scenario, the control energy does not exceed the smallest eigenvalue of the Gramian matrix, which is denoted as $\lambda _{max}(\textbf{W}_\text {B}(t_\text {f}))$^47^. This study evaluates the required control energy of a given input set by examining the average control energy towards all possible target states. We use $\text {Trace}({\textbf{W}_\text {B}(t_\text {f})})$ instead of $\text {Trace}({\textbf{W}_\text {B}(t_\text {f})}^{-1})$ as a measure of control energy for two main reasons: first, $\text {Trace}(\textbf{W}_\text {B}(t_\text {f})^{-1})$ and $\text {Trace}(\textbf{W}_\text {B}(t_\text {f}))$ satisfy a relation of inverse proportionality^48^, $\text {Trace}(\textbf{W}_\text {B}(t_\text {f})^{-1}) > N^2/\text {Trace}(\textbf{W}_\text {B}(t_\text {f}))$, so that the information obtained from the two metrices are correlated with one another and, second, ${\textbf{W}_\text {B}}(t_\text {f})$ is typically ill-conditioned and close to singularity, so $\text {Trace}(\textbf{W}_\text {B}(t_\text {f})^{-1})$ cannot be accurately computed even for small brain networks. Therefore, we use

3 $$\begin{aligned} \varepsilon (t_{\text {f}}) = \text {Trace}({\textbf{W}_\text {B}(t_\text {f})}), \end{aligned}$$

to evaluate the control energy of brain networks, with higher values indicating that less energy is required to move around the state space in all directions. However, calculating control energy using $\text {Trace}({\textbf{W}_\text {B}(t_\text {f})}^{-1})$ or $\lambda _{max}(\textbf{W}_\text {B}(t_\text {f}))$ is a challenging task and developing an approach to reduce energy by these metrics leads to an NP-complete problem^34^. Additionally, these metrics do not offer any insight into why there may be high or low control energy for a given set of input nodes. Rather, they only provide a numerical evaluation of control cost.

It has been shown that control energy can be evaluated by the structural parameter longest control chain (LCC), i.e., the longest distance between non-input nodes and the closest input to them

4 $$\begin{aligned} \text {LCC} = \max _{w\,\in\, \mathcal {V}}\min _{v \in \mathcal S} d(v,w), \end{aligned}$$

where $\mathcal S$ is the set of input nodes and *d*(*v*, *w*) is the length of the shortest path connecting from *v* to *w*. It is proven that any change in LCC can significantly reduce or increase the energy necessary to control networks^35–37,49^. Figure 1 shows an illustrative example of how LCC affects the control energy in a small network. See reference^35^ for more details about the role of LCC in the controllability of complex networks. Using this structural parameter, we can gain insight into the relationship between the energy required to control brain networks and the architecture of white matter fibers. It also helps us understand the role of different brain regions in various control schemes.

**Figure 2:**
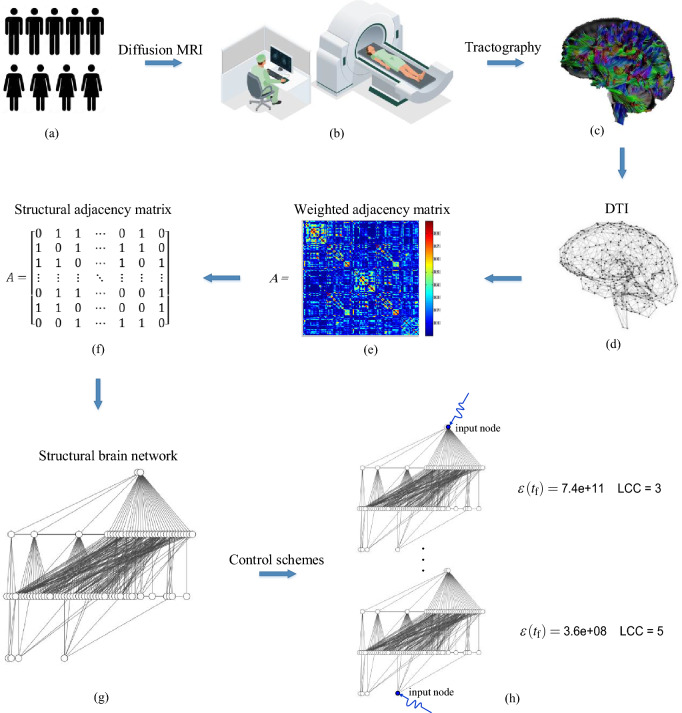
The schematic of the work process. **(a–c)** We conducted a study on the controllability of structural brain networks using DTI scans on seven healthy individuals of varying genders and ages. DTI is a non-invasive imaging technique that illustrates how white matter, which is the highways of our brain, connects different regions of the brain. The AAL atlas determines these regions. **(d–e)** The DTI result is a weighted adjacency matrix that displays the strength and connections between regions. **(f)** To study the structural controllability of brain networks, we require the structural adjacency matrix, which is a zero-one matrix that focuses on the presence or absence of connections. We substitute any element $a_{ij}>0$ with one to obtain the structural adjacency matrix. **(g)** Structural brain network constructed from the adjacency matrix in **(f)**. The brain network obtained is undirected. Any undirected link (*i*, *j*) can be considered a bidirectional link with two connections: one from *i* to *j* and the other from *j* to *i*. Since we study control theory on directed networks, we substitute any undirected link $(i-j)$ in the network with two directed links $(i \rightarrow j)$ and $(j \rightarrow i)$. **(h)** Finally, we analyze different control schemes.

In this study, we model human brains through networks, in which nodes represent regions while links show white matter fibers connecting them. Investigating the dynamic and required control energy in brain networks would be best done by having access to the adjacency matrix $\textbf{A}$ with exact weights. However, if we lack precise information about the weights, it is still possible to gain insight into the dynamic and control costs of the brain networks based on their structure. We used structural controllability and structural parameter LCC, which is independent of the weights. LCC measures the distance between the inputs and other nodes in the network and is affected by the presence or absence of a link rather than its weight. Therefore, our research focuses on the structural controllability of brain networks using the input node placement and its impact on the LCC. For this, we require three parameters: the structural adjacency matrix $\textbf{A}$, input matrix $\textbf{B}$, and the control time $t_\text {f}$. We constructed structural brain networks from the weighted adjacency matrix obtained by DTI to investigate their structural controllability. The process of constructing the weighted adjacency matrix of brain networks, and then the structural matrix, will be explained in the next section. The input matrix $\textbf{B}$ can be created by setting the node index of the input nodes to one. Additionally, we consider the control time as $t_\text {f} = 1$. Please refer to Fig. 2 for a schematic of our work process.

### Data acquisition and brain network construction

Diffusion tensor imaging (DTI) is an MRI technique used to estimate the white matter organization of the brain by utilizing anisotropic diffusion. In this method, sets of neurons are assigned to regions with varying total numbers. Automated Anatomical Labeling (AAL)^50^, an atlas of the human brain that assembles neurons into 90 regions, is used in this study. Thus, the network nodes in this study represent regions of the AAL atlas. This architecture considers the anatomical characteristics of brains^50^ and, as consistent with prior works^28^, leads to an adjacency matrix $\textbf{A} \in R^{90 \times 90}$. After processing the outcome of DTI, the result obtained is a weighted adjacency matrix. Therefore, the weighted adjacency matrix $\textbf{A} \in R^{90 \times 90}$ illustrates the extracted estimations of links between 90 regions and their strength while regions are mapped to the AAL atlas. More details about the data collecting setup and parameters for DTI are provided in “Methods” section. To obtain the structural adjacency matrix $\textbf{A}$, we change the weighted adjacency matrix to a binary matrix by setting each element $a_\text {i j} > 0$ to one. The corresponding structural adjacency matrix focuses on the brain network connections. Additionally, this adjacency matrix represents the brain in a symmetric structure, which provides information about the links, not their directions. However, any unidirectional link between two nodes *i* and *j*, $(i -j)$, could be considered a bidirectional link in such a way that there is a connection from *i* to *j*, $(i \rightarrow j)$, and vice versa from *j* to *i*, $(j \rightarrow i)$. Since we utilize the control theory framework on directed networks, we considered this interpretation to construct directed brain networks.

## Methods

In this work, we considered seven healthy human adults of different ages and genders to investigate their brain networks. As mentioned in the “Data acquisition and brain network construction” section, we first use the MRI technique to scan brains. Data collecting setups and parameters are as follows.

### Image acquisition

MRI images were acquired on a Siemens 3.0 Tesla scanner (Prisma, Erlangen, Germany), at the Iranian National Brain Mapping Lab (NBML) (www.nbml.ir). A few characteristics of this machine included 50-cm FOV with the industry best homogeneity; whole-body; superconductive zero helium oil-off 3T magnet; and head/neck 20 direct connect. We used the 64-channel head coil in our study. The MRI protocols were selected to match the international projects, such as the UK Biobank or the ENIGMA consortium. Using an EPI Diffusion weighted protocol, 66 volumes were acquired with the imaging parameters of TR = 9900 (ms), TE = 90 (ms), b-value= 0 (s/$\text {mm}^2$), b-value = 1000 (s/$\text {mm}^2$), diffusion directions = 64; TA= 11:05 min, voxel size = $2.0\times 2.0\times 2.0$ (mm), FOV read =256 (mm); number of slices= 65; distance factor = 0; phase encoding direction= anterior $>>$ posterior, matrix size = $128 \times 128 \times 65$. Using an MPRAGE protocol, T1-weighted structural scans were acquired for clinical diagnostic purposes, with the imaging parameters of TA = 4:12 min, TR = 1800 (ms), TE = 3.53 (ms), TI = 1100 ms, flip angle = 7 degrees, voxel size = $1.0\times 1.0\times 1.0$ (mm), multi-slice mode = sequential, FOV read = 256 (mm), number of slices = 160, phase encoding direction = anterior>> posterior, matrix size = $256\times 256\times 160$.

### DWI data analysis method

DWI data analysis and tractography were performed with ExploreDTI software , University Medical Center, Utrecht, the Netherlands in the following order:

1. *DWI data pre-processing* (i) data conversion from DICOM formats to NIfTI and Bmatrix text-file formats; (ii) the right-left orientation was checked using the flip/ permute plugin; (iii) conversion to MAT files; (vi) the orientation of the MAT files was checked using the glyph plugin; (v) correction for subject motion an and eddy current/EPI distortions using a cubic spline interpolation.
2. *Tractography* We use a deterministic streamline method to obtain fibers, by setting the Fractional Anisotropy (FA) threshold to 0.2, the angular threshold to 30 (degree), linear interpolation method and fiber length range 50–500 (mm).
3. *Connectivity matrix* For network analysis, we use an atlas template/labels method that obtains connectivity matrics (CMs) from AAL90 atlas^50^; for more information and example studies about the network analysis tools of ExploreDTI see the work of Reijmer et al.^51–53^. We use CMs for the number of tracks in graph analysis.

We have shown the network properties of studied human brains in Table 1 in which $|\mathcal {V}|$ and $|E|$ indicate the number of network nodes and links, respectively. The data we observe from DTI shows a small number of isolated nodes in brain networks, consistent with previous works^27^. We determined these regions in each network, shown in the column $R_\text {isolated}$ of the Table 1. There are structural network properties that affect both network controllability and control energy. For example, we have shown previously that network density and the degree of heterogeneity are two network properties with significant impact on LCC^35^. In principle, the heterogeneity of a network measures the diversity in the node degrees with respect to a completely homogeneous network of the same number of nodes. We calculate the heterogeneity of nodes based on the maximum heterogeneity of input degree and heterogeneity of output degree $H = max(H^{in}, H^{out})$ where $H^{in/out}$ can be defined by $H_{in/out}=\frac{1}{rN^2}\sum _i \sum _j \bigg (k_i^{in/out}-k_j^{in/out}\bigg )$^14^. Here, *r* is a constant, and $k_i^{in/out}$ shows the input/output degree of node *i*. We determined these network properties in each studied network that columns *c*, $K_\text {min}$, $K_\text {max}$, and *H* show the average degree, the minimum and the maximum degree in the network, and the degree of heterogeneity, respectively. The result shows that brain networks are dense, and the degree of network nodes could be varied by a large difference. Note that in brain networks, the out-degree and in-degree are equal for each region, $K^\text {out} = K^\text {in}$. Since LCC points to the distance between inputs to other network nodes, we also calculated the average distance between network nodes and column *d* shows the result.

**Table 1:** The properties of studied brain networks.

Networks	Age	Gender	$|\mathcal {V}|$	$|E|$	$R_\text {isolated}$	*H*	*c*	$K_\text {min}$	$K_\text {max}$	*d*
1	50	F	90	1251	Amygdala R	0.61	13.9	1	35	2.1
2	66	F	90	1183	Amygdala L–Heschl R–Rolandic Oper L	0.54	13.1	1	25	2.3
3	21	M	90	1091	Amygdala R	0.62	12.1	2	33	2.2
4	64	F	90	1086	–	0.57	12.0	1	26	2.3
5	47	M	90	1111	Amygdala R–Amygdala L–Heschl L	0.70	12.3	2	37	2.2
6	66	M	90	1120	Amygdala L	0.61	12.4	1	43	2.2
7	35	M	90	1090	Amygdala R	0.61	12.1	1	30	2.3

## Results

### The structural controllability of studied brain networks

We used constructed brain networks to analyze the structural controllability of human brains concerning the parameter LCC. For this purpose, we first determined the minimum number of input nodes necessary to ensure the structural controllability of each network. In this regard, we eliminated isolated nodes that do not affect network connections and focused on the connected graph. Note that our primary goal is to establish a connection between the control energy of brain networks and their structure using the parameter LCC. Isolated nodes in the network have no connections to other nodes and, therefore, do not affect the LCC. Although considering isolated nodes increases the number of input nodes required to ensure controllability, it does not have a significant impact on the control energy. Furthermore, taking them into account does not reveal any information about the relationship between the architecture of white matter fibers and the control energy. We then constructed the associated bipartite graphs of brain networks and utilized the maximum matching algorithm to determine the minimum number of control signals required to ensure structural controllability. We found that all nodes were matched after implementing the algorithm, indicating that only one control signal is needed to steer the network’s state to any desired one, $N_\text {i} = max (1, N_\text {u}) = 1$.

Next, we considered each region as an input node and evaluated the smallest eigenvalues of the controllability Gramian matrix. The results showed values higher than zero, indicating that the system can theoretically be controlled through any brain region. Therefore, in theory, any brain region can be driven as an input node to move the initial state of brain networks into any desired state. This result is consistent with previous studies showing that brain networks require only an input node to be controllable^25,26^.

### Using a single input node and the impact of LCC on the control energy

We now explore the role of different regions in controlling brain networks, focusing on the required control energy and their LCC. Firstly, we identify regions with the lowest and highest required energy to control the studied brain networks. All control energies will be measured by $\varepsilon (t_\text {f}) = \text {Trace}({\textbf{W}_\text {B}(t_\text {f})})$ which means that a smaller value will require more control energy, on average, to control the network. Additionally, all values obtained for $\varepsilon (t_\text {f})$ will be presented in $\log _{10}{\varepsilon (t_\text {f})}$ format. The Table 2 displays regions that control brain networks with the minimum and maximum required energy, the exact value of $\log {\varepsilon (t_\text {f})}$, and their LCC. Our results indicate that the required control energy in analyzed brain networks can vary based on the selected input node, with a difference of up to 3.6 in $\log {\varepsilon (t_\text {f})}$. Furthermore, it shows that regions that control brain networks with the minimum energy have LCC = 3, while regions that require the highest cost for controllability have LCC = 5. We determined the smallest and largest achievable LCC in brain networks to evaluate this outcome. The columns $\text {LCC}_{\text {min}}$ and $\text {LCC}_{\text {max}}$ in Table 2 show the smallest and largest possible LCC in controllability of studied brain networks with one input node, which is three and five, respectively. In other words, regions that require the highest control energy in the controllability of brain networks have the largest possible LCC, while those that control the networks with the lowest control energy have the smallest LCC.

**Table 2:** Regions that control brain networks with the lowest and highest energy.

Network	The minimum control energy				The maximum control energy				$\text {LCC}_{\text {min}}$	$\text {LCC}_{\text {max}}$
	$\text {Input node}$		$\log {\varepsilon (t_\text {f})}$	$\text {LCC}$	$\text {Input node}$		$\log {\varepsilon (t_\text {f})}$	$\text {LCC}$		
	node	region			node	region				
1	2	Frontal Sup L	11.8	3	78	Heschl L	8.2	5	3	5
2	3	Frontal Sup R	9.8	3	78	Heschl L	7.2	5	3	5
3	3	Frontal Sup R	10.1	3	62	SupraMarginal L	6.8	5	3	5
4	72	Putamen L	9.2	3	16	Rolandic Oper L	6.2	5	3	5
5	73	Putamen R	11.6	3	44	Cuneus L	8.7	5	3	5
6	66	Precuneus L	10.3	3	10	Frontal Inf Oper L	6.7	5	3	5
7	37	Hippocampus R	9.6	3	20	Olfactory L	6.9	5	3	5

**Figure 3:**
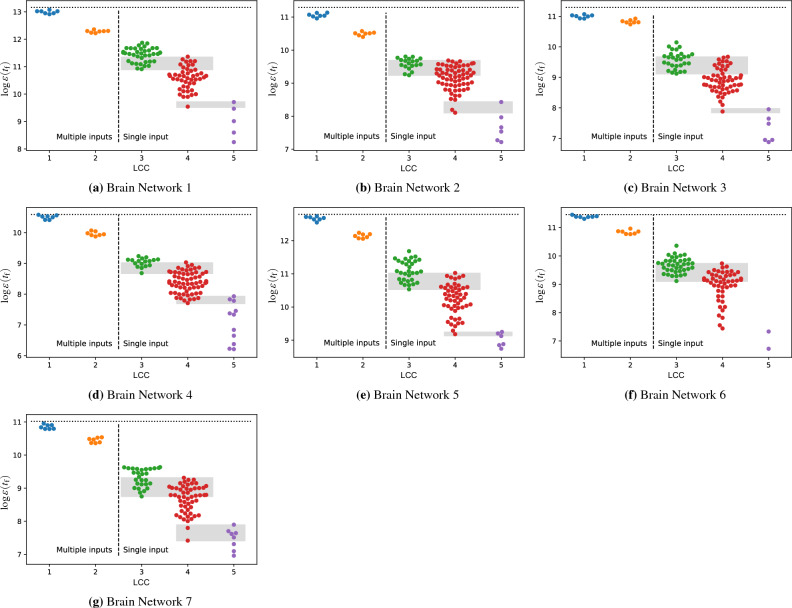
The relationship between control energy and LCC in brain networks. The right side of the vertical dashed line shows the energy distribution of controlling brain networks with a single input where the LCC is between three and five. Considering that high control energy is related to a low value of $\log {\varepsilon (t_\text {f})}$, the result indicates that brain regions with the highest control energy correspond to nodes with LCC = 5, nodes with LCC = 4 contribute in the middle range of energy, and the lowest control energies are observed in regions with LCC = 3. However, there is an overlap between the control energy of brain regions with LCCs 3 and 4 and similarly between regions with LCCs 4 and 5. This overlap, shown by the grey colour, is expected given the high density of brain networks, the small difference between LCCs, and the impact of other parameters on control energy. The left side of the vertical dashed line indicates the energy distribution of controlling brain networks with smaller LCCs using multiple input nodes. The illustrated energies are associated with input sets that control the brain networks with LCC equal to 1 or 2 with the minimum number of input nodes. Note that using more input nodes requires less energy to control networks. However, in real-world scenarios, limitations restrict the number of inputs that can be used. The horizontal dotted line at the top of the figures represents the required $\log {\varepsilon (t_\text {f})}$ to control brain networks when all nodes are considered input nodes. The result indicates that by reducing the LCC in the controllability of brain networks, the energy needed for control is reduced effectively. Specifically, by considering a small portion (almost ten percent according to the Table 3) of nodes as inputs to control brain networks with LCC = 1, we can attain a required energy equivalent to the control energy when all brain regions are input nodes.

We then conducted a comprehensive assessment of various regions of the brain instead of focusing solely on those with the highest or lowest control energy. We considered each region as an input node to define different control schemes and evaluated their control energies and LCCs. In Fig. 3, we present the energy distribution needed to control brain networks with different input nodes and their corresponding LCCs. The vertical axis represents $\log {\varepsilon (t_\text {f})}$, and the horizontal axis shows the LCC. The data shows that an increase in the LCC leads to a reduction in $\log {\varepsilon (t_\text {f})}$, which means more energy is needed to control brain networks. There might be some overlap between the energy required for regions with LCC values of 3 and 4, as well as between regions with LCC values of 4 and 5. It is important to note that while LCC has a dominant impact on the control energy, other factors also contribute to determining it. Due to the high density of brain networks, the use of only one input node, and the effect of other parameters, we expected some overlap in energy requirements for regions with LCC values of 3 and 4 and regions with LCCs of 4 and 5. However, the results indicate that LCC is an effective determinant of the required energy scale in brain networks. The minimum energy is associated with regions with LCC = 3, while in the middle range of energy, regions with LCC = 4 contribute, and regions with LCC = 5 require the maximum control energy. To analyze the results statistically, we used the Wilcoxon rank-sum test. This test is commonly used to compare two groups of nonparametric data that are not normally distributed. Its purpose is to test the null hypothesis that two populations possess the same distribution, indicating no significant difference between them^?,?^. To perform this analysis, we compared the data for each LCC = $\ell$ and $\ell + 1$ in Fig. 3 as two groups. Specifically, we evaluated the test for data in LCC = 1 vs 2, LCC = 2 vs 3, LCC = 3 vs 4, and LCC = 4 vs 5. The p-values for the considered group of samples (for the average of seven studied brain networks) are $6.7\text{e}{-3}$, $2.7\text{e}{-4}$, $5.5\text{e}{-8}$, and $1.3\text{e}{-3}$, respectively. These values indicate that the null hypothesis can be rejected. The variances of the p-values are negligible, so we have omitted them.

We have identified brain regions with consistently low LCC across at least six out of seven studied networks that control the networks with the lowest energy. The regions we have identified include the Precentral, Frontal Sup, Precuneus, Paracentral Lobule, Frontal Sup Orb, Supp Motor Area, Frontal Sup Medial, Insula, Hippocampus, Putamen, and Parietal Sup regions. The Superior Frontal and other association regions were among them, consistent with their known role as major connectivity hubs of the brain that support integrated function^54,55^. We analyzed these regions and found that they are high-degree nodes, with an average degree of 21.2 and a standard deviation of 6. This outcome may help to shed light on the structural explanation of why highly connected brain regions, most of which have a small LCC (LCC = 3), are known as regions that, on average, can move the brain around the state space with a little effort^26^. On the other hand, we have identified several regions that are the strongest contributors to the highest control energy. These regions include the Frontal Mid Orb, Rolandic Oper, Olfactory, Amygdala, SupraMarginal, and Heschl, which are primary regions in the auditory and visual cortex. This is consistent with the idea that their connectivity is more local and specialized^56^, thus less well-positioned to trigger global state changes. These regions have an average degree of 1.8 with a standard deviation of 0.8, making them the lowest-degree nodes in brain networks.

### Using multiple input nodes to lower the LCC

Controlling brain networks is a challenging task in practice, despite the theoretical possibility of controlling them through a single control region^26,30^. Although controlling brain networks with a single input can be more energy-efficient by selecting highly connected nodes that lead to lower LCCs, it still may require a large amount of energy for real implementations. A potential solution to decrease control energy could be considering more input nodes. However, determining the exact number of nodes required and their proper locations can be challenging.

Our research aims to reduce the energy required to control brain networks by utilizing the impact of LCC on control energy. Specifically, we want to identify input nodes that can decrease LCCs in the controllability of brain networks. However, determining the minimum number of inputs and their location to ensure a given LCC is still a challenging problem that is not easily solvable^35^. For this purpose, we use the algorithm suggested in our previous work, which can determine the minimum input nodes required to reach a given LCC in the controllability of networks^35^. In some cases, this algorithm can find the exact solution, otherwise it finds an input set close to the optimal answer. We use the notation $N_\text {i}(\ell )$ to represent the minimum number of input nodes required to control brain networks with LCC = $\ell$. Our findings for the required number of inputs to control studied brain networks with smaller LCCs = $\ell$ ($\ell$ = 1 and 2) are presented in column $n_\text {i}(\ell )$ of the Table 3, where $n_\text {i}(\ell ) = N_\text {i}(\ell ) / N$.

Considering the effect of highly connected nodes, we also used a naive and greedy method that involves adding the node with the highest degree to the input set until reaching the desired LCC. We sorted the nodes in each network according to their degree in descending order and added nodes to the input set from the first of this list until LCC = $\ell$ is reached. We represent the number of nodes that are determined by this greedy approach to control brain networks with LCC = $\ell$ by the notation $N_\text {i}^\text {hubs}(\ell )$, and its results for our studied brain networks are shown in column $n_\text {i}^\text {hubs}(\ell )$ of Table 3, where $n_\text {i}^\text {hubs}(\ell ) = N_\text {i}^\text {hubs}(\ell )/N$.

Our findings indicate that we need approximately 2% of network nodes to control brain networks with LCC = 2 and almost ten percent to reach LCC = 1. The results also show that selecting only highly connected input nodes is not an effective approach to reducing the LCC in analyzed brain networks. There is a considerable difference, up to six times larger in the worst case and equal to 56% of network nodes, between the number of input nodes required to control brain networks with LCC = $\ell$ using the two approaches.

**Table 3:** The number of input nodes required to control brain networks with lower LCCs = $\ell$ ($\ell$ = 1 and 2).

Network	LCC = 2		LCC = 1	
	$n_\text {i}(2)$	$n_\text {i}^\text {hubs}(2)$	$n_\text {i}(1)$	$n_\text {i}^\text {hubs}(1)$
1	0.02	0.03	0.10	0.33
2	0.02	0.06	0.11	0.31
3	0.02	0.10	0.08	0.21
4	0.02	0.07	0.12	0.52
5	0.02	0.04	0.11	0.26
6	0.02	0.06	0.11	0.67
7	0.02	0.08	0.08	0.37

The column titled $n_\text {i}^\text {hubs}(\ell )$ indicates the fraction of required input nodes obtained by the greedy approach to control brain networks with LCC = $\ell$, while $n_\text {i}(\ell )$ shows the fraction of the optimal number of required inputs.

**Table 4:** The degree of nodes in the optimal input set, i.e., the input set with the minimum number of nodes, to reduce the LCC in the controllability of brain networks.

Network	LCC = 2	LCC = 1
	*c*	*c*
1	[7, 20]	[5, 15, 16, 17, 19, 20, 23, 29, 30]
2	[13, 23]	[1, 2, 9, 14, 15, 16, 19, 20, 24, 24]
3	[10, 31]	[2, 13, 17, 17, 20, 31, 32, 33]
4	[17, 26]	[1, 10, 11, 11, 12, 12, 14, 15, 23, 23, 24]
5	[3, 26]	[5, 8, 13, 14, 14, 16, 17, 23, 28, 31]
6	[10, 25]	[3, 3, 9, 13, 21, 22, 23, 24, 25, 42]
7	[9, 23]	[3, 14, 16, 17, 18, 24, 25, 26]

The optimal input set is not unique, and analyzing various input sets shows similar results but different proportions of nodes having low or high degrees.

Our study has shown that the energy-efficient way to control brain networks may not necessarily involve using the network hubs as input nodes. We have analyzed input sets that can control studied brain networks with smaller LCCs using the least number of input nodes. We have represented the degree of these input nodes using the variable *c* in the Table 4, and sorted the degrees in ascending order to simplify the analysis of their distribution. Based on our analysis, we recommend incorporating nodes with low degrees, such as *c* = 1, 2, 3, and 5, nodes with degrees similar to the average degree in brain networks, such as *c* = 10, 11, 14, and 16, as well as high-degree nodes like *c* = 30, 33, and 42 to reduce the LCC with the smallest number of input nodes. It is important to note that there is no one specific input set that can ensure structural controllability while also meeting the constraint on LCC. Analyzing various input sets shows that the results are consistently similar but with different proportions of nodes having low or high degrees.

In the examined brain networks, nodes with high degrees of connectivity are almost connected to a third of the network’s nodes. This means that in controlling brain networks with a single input, driving a high-degree node can lead to fewer hops between the input node and other network nodes, resulting in lower LCCs and less control energy. The analysis of studied brain networks also shows that there is assortativity among high-degree regions. This means that highly connected nodes tend to connect to other nodes with similar properties. This human connectome’s rich-club organization refers to densely interconnected brain network hubs^57,58^. Therefore, adding only highly connected nodes to the input set is not an effective solution when employing multiple inputs to control brain networks with lower energy. This is because it does not efficiently reduce the distance between current inputs and other network nodes, especially those on a longer distance, which is necessary to lower the LCC. To lower the LCC, it is important to distribute the input nodes throughout the network. Table 5 provides information about the distance between input nodes using two various approaches. The column $d_i(\ell )$ shows the average distance between nodes in the optimal input set with the minimum size to control brain networks with LCC = $\ell$. On the other hand, $d_i^\text {hubs}(\ell )$ represents the average distance between nodes in an input set of the same size, but which only includes high-degree nodes. To create this input set, we sorted the nodes in each network by their degree in descending order and added the nodes from the top of this list to the input set until we reached the same size as the optimal input set. Additionally, the ’std’ column indicates the standard deviation of the mean distances. According to the table, nodes in the optimal input set have a mean distance of approximately $\ell + 1$ to ensure LCC = $\ell$. However, highly connected nodes are usually located close to each other. For example, $d_i(2)$ shows that optimal input nodes to control brain networks with LCC = 2 are all located throughout the network with a distance of three from each other. While $d_i^\text {hubs}(2) = 1$ with a standard deviation of zero shows that all high-degree nodes with the same size are direct neighbours of each other. This is why we need 2–6 times more inputs, compared to the minimum number of required nodes in the Table 3, to control brain networks with smaller LCCs using only high-degree nodes.

**Table 5:** Comparing the distance between nodes in the optimal input set and the input set including only high-degree nodes.

Network	LCC = 2				LCC = 1			
	$d_\text {i}(2)$	std	$d_\text {i}^\text {hubs}(2)$	std	$d_\text {i}(1)$	std	$d_\text {i}^\text {hubs}(1)$	std
1	3	0	1	0	1.8	.2	1.3	.1
2	2	0	1	0	2.5	.4	1.7	.1
3	2	0	1	0	2.1	.3	1.3	.2
4	3	0	1	0	2.3	.3	1.5	.1
5	3	0	1	0	2.1	.2	1.1	.0
6	3	0	1	0	2.3	.4	1.4	.1
7	3	0	1	0	2.1	.3	1.5	.1

The column labeled $d_i(\ell )$ displays the average distance between nodes in the optimal input set. This input set is the smallest possible set that can control the brain network with a LCC = $\ell$. On the other hand, $d_i^\text {hubs}(\ell )$ represents the average distance between nodes in the input set that has the same size as the optimal input set but includes only high-degree nodes. Additionally, the “std” column indicates the standard deviation of the mean distances.

We have calculated the required energy to control brain networks with the least input nodes that ensure LCC = 2 or 1 in Fig. 3. As shown, reducing the LCC decreases the required energy in the controllability of brain networks. Note that there is a trade-off between the number of input nodes and the control energy. More nodes as inputs mean less energy is needed to control the network, but due to constraints in real implementations, a limited number of inputs can be used. The horizontal dotted line in Fig. 3 represents the energy required to control brain networks when all the regions are considered input nodes. Our finding demonstrates that by reducing the LCC to 1 by almost ten percent of brain regions as input nodes, we can control brain networks with similar energy as when all regions are inputs.

We analyzed one hundred different input sets to identify regions that have the most participation in controlling brain networks while adhering to the constraint LCC = 1. Regions that participated in all input sets play a primary role in reducing the LCC and energy in controlling brain networks. Identifying these regions could provide valuable insights into controlling brain networks with less energy. The result is presented in Table 6. Moreover, our analysis of repeated regions in different brain networks revealed that Precuneus is the most frequently repeated region, appearing in five out of seven studied brain networks, as the primary contributor.

**Table 6:** Brain regions with high frequency in input sets that control studied brain networks with LCC = 1.

Networks	Regions
1	Precuneus L, Frontal Inf Tri L, Frontal Med Orb L, Temporal Pole Sup L, Temporal Pole Sup R
2	Precuneus L, Temporal Mid L, Calcarine R, Temporal Pole Mid R
3	Precuneus L, Temporal Mid L, Frontal Sup R, Frontal Sup Medial L, Lingual L
4	Precuneus L, Hippocampus R, Occipital Mid L, Postcentral R, Parietal Inf L
5	Frontal Sup Orb L, Temporal Inf R
6	Precuneus L
7	Insula L, Cingulum Ant L, Hippocampus R, Fusiform L

Finally, we studied the role of the degree distribution using network models where we fixed the expected degree of each node, but their connections are random. To reveal the role of higher-order structural properties, such as degree correlations or community structure^59–62^, we first measure $n_\text {i}(\ell )$ for our studied brain networks, and then randomize the networks while preserving their degree distribution but otherwise randomly rewiring their links, thus removing any higher-order structure. We also prevented the rich-club organization in the rewired networks. For this purpose, we chose a link between two hubs and a link between two small-degree nodes and then rewired them to remove the assortativity among hubs. Finally, we measure $n_\text {i}^\text {rand}(\ell )$, the fraction of required input nodes needed to control these randomized networks. Table 7 compares $n_\text {i}(\ell )$ to its randomized counterpart $n_\text {i}^\text {rand}(\ell )$: If the degree sequence of a network would completely determine the number of control signals, the two values would be equal, any difference is due to the structural features removed by randomization. We have observed a correlation between $n_\text {i}(\ell )$ and $n_\text {i}^\text {rand}(\ell )$, indicating that the degree distribution plays an important role in determining required input nodes in the controllability of brain networks. Higher-order network properties such as degree correlations and community structure are shown to play a limited role. We also noticed that as $\ell$ decreases, the correlation becomes slightly weaker. For LCCs equal to 5, 4, and 3, the results are almost the same, while for LCC = 2, they differ slightly in decimals that we have not taken into account due to insignificance. Finally, for LCC = 1, the results show a small difference. We considered the average of twenty randomized networks for each $n^\text {rand}_\text {i}(\ell )$, and for each randomization, we performed $(|E|/2) \times \ln (1/\epsilon )$ number of link rewiring trials, where $\epsilon$ lies between $10^{-6}$ and $10^{-7}$, as suggested by Ref.^63^. The standard deviations of averages are small, so we have ignored them in our presentation.

**Table 7:** Minimum number of inputs $n_\text {i}(\ell )$ in model networks.

Network	LCC = 5		LCC = 4		LCC = 3		LCC = 2		LCC = 1	
	$n_\text {i}(5)$	$n_\text {i}^\text {rand}(5)$	$n_\text {i}(4)$	$n_\text {i}^\text {rand}(4)$	$n_\text {i}(3)$	$n_\text {i}^\text {rand}(3)$	$n_\text {i}(2)$	$n_\text {i}^\text {rand}(2)$	$n_\text {i}(1)$	$n_\text {i}^\text {rand}(1)$
1	0.01	0.01	0.01	0.01	0.01	0.01	0.02	0.02	0.10	0.09
2	0.01	0.01	0.01	0.01	0.01	0.01	0.02	0.02	0.11	0.10
3	0.01	0.01	0.01	0.01	0.01	0.01	0.02	0.02	0.08	0.09
4	0.01	0.01	0.01	0.01	0.01	0.01	0.02	0.02	0.12	0.10
5	0.01	0.01	0.01	0.01	0.01	0.01	0.02	0.02	0.11	0.08
6	0.01	0.01	0.01	0.01	0.01	0.01	0.02	0.02	0.11	0.09
7	0.01	0.01	0.01	0.01	0.01	0.01	0.02	0.02	0.08	0.09

### The impact of the number of paths between brain regions

In the previous sections, we showed how LCC affects the required control energy. However, Fig. 3 demonstrates that the range of control energy in brain networks varies, regardless of the effect of LCC and the selected input nodes. For instance, $\log {\varepsilon (t_\text {f})}$ to control Brain Network 4 with a single input is almost $6< \log {\varepsilon (t_\text {f})} < 9$, while for Brain Network 5, it is approximately $9< \log {\varepsilon (t_\text {f})} < 12$. Considering that a lower value of $\log {\varepsilon (t_\text {f})}$ is correlated with higher control energy, it means that controlling Brain Network 5 requires less energy than controlling Brain Network 4, regardless of the selected input nodes and the effect of LCC. Therefore, this section aims to investigate other structural features that affect the control energy in brain networks beyond LCC. For example, previous studies have revealed that increasing the number of paths connecting inputs and non-input nodes reduces the control energy^37^. However, this factor may be overlooked in our LCC-based analysis when the number of paths between the input and non-input nodes differs in two control schemes with the same LCC.

**Figure 4:**
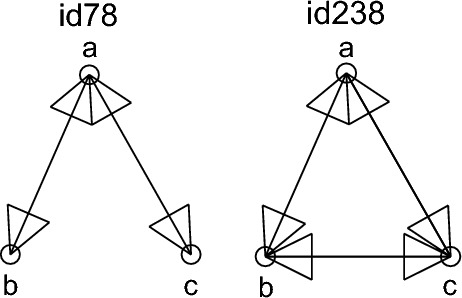
All possible subgraphs with three nodes in brain networks.

Our goal in this section is to study the control energy of brain networks by examining the number of paths between regions in their structure. However, determining all possible simple paths between two given nodes in a graph is an NP-hard problem. Its complexity is O(*N*!), which makes it difficult to calculate the number of paths from input nodes to other regions in the brain networks. To address this issue, we use motifs—recurrent and statistically significant subgraphs or patterns of a larger graph^64^–to assess the number of paths between regions. In collected brain networks, we observe two motifs (id78 and id238) that are shown in Fig. 4 as possible 3-node motifs in brain network structures. By increasing the number of these patterns in the network, input nodes can access other regions through more separate paths. For instance, two regions, a and c, in the 3-node motif id238 can reach each other either through the direct link (a,c) or through the third region b and the path (a,b), (b,c). Therefore, in the following, we will investigate the control energy in brain networks concerning the number of motifs in their structures.

**Figure 5:**
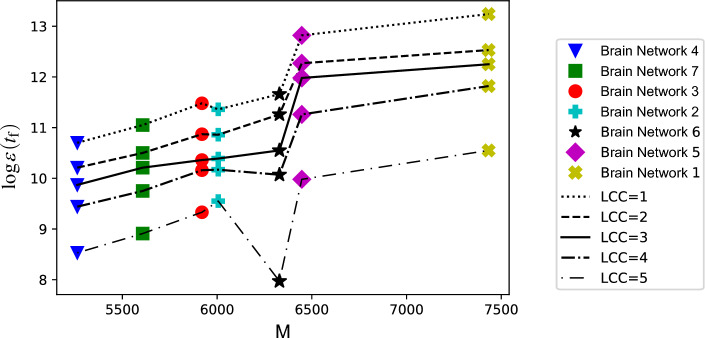
The number of motifs in the structure of analyzed brain networks and its effect on required control energy. The number of motifs *M* is shown by the horizontal line, and the vertical dashed line displays $\log {\varepsilon (t_\text {f})}$. The results indicate that an increase in the number of motifs generally leads to lower energy requirements for controlling brain networks. The analysis has been conducted separately for regions with the same LCCs (LCC = 1, 2, 3, 4, and 5). There is an exception in LCC = 5 and Brain Network 6, which could be due to the existence of only two regions with this value of LCC in this network. Please note that a lower value of $\log {\varepsilon (t_\text {f})}$ is correlated with higher control energy.

We have conducted a study on brain networks and counted the number of motifs (id78 and id238) in their structure. The results are presented in Table 8, where the number of motifs is shown by *M* and the data is sorted in descending order. We then analyzed the average control energy of regions with the same LCC in the networks, as shown in Fig. 5. The result shows that controlling brain networks requires varying amounts of energy, even with the same LCC. As the number of motifs in brain networks increased, the energy needed to control decreased, revealing that the number of paths between regions in the brain networks has an impact on control energy. Therefore, the higher the number of these patterns, the lower the energy required to control analyzed brain networks.

**Table 8:** The number of motifs id78 and id238 in the studied brain networks.

Network	*M*
1	7433
5	6447
6	6329
2	6006
3	5920
7	5607
4	5262

The number of motifs is denoted by *M*. Brain networks are ranked in ascending order based on the number of motifs in their structure, with Brain Network 1 having the highest number of motifs and Brain Network 4 having the lowest.

## Conclusion

The controllability of brain networks involves the ability to drive certain brain regions, known as input nodes, using external signals to steer the initial neurophysiological activity of the brain toward a desired one. To evaluate whether it is possible to control a network in real-world situations, it is crucial to determine the amount of energy required for control, and the energy needed to control brain networks varies depending on the regions selected as input nodes. Leveraging our prior work on input node placement and its impact on the longest control chain (LCC), we highlight the role of white matter fiber architecture in the energy required to control brain networks. By understanding how brain structure affects its dynamic and controllability, we can design more energy-efficient control strategies.

We show that controlling brain networks using a single input can be more energy-efficient by selecting highly connected regions as input nodes that lead to lower LCCs. Yet, using only one input node may still require high energy to control brain networks, and one approach is using multiple input nodes to reduce the needed energy. However, we can only employ a restricted number of input nodes to control a system in real-world applications due to practical constraints. Our research employs the impact of LCC on control energy to identify the minimum number of input nodes required to control brain networks with smaller LCCs. The obtained result indicates that decreasing the LCC can effectively reduce the control energy in brain networks. Specifically, by driving only a small fraction (around 10%) of network nodes as inputs to reach the smallest possible LCC, we can effectively control the studied brain networks with the same amount of energy when all nodes act as inputs. We indicate that only focusing on high-degree regions is not an efficient solution to decrease the control energy in brain networks. The minimum number of input nodes to control brain networks with lower energy is a combination of regions with both low and high degrees, not just hubs. This is because of the human connectome’s rich-club organization, which means brain network hubs are densely interconnected. As a result, merely selecting hubs as inputs does not properly reduce the distance between inputs and other network nodes, which is necessary to decrease the LCC and control energy.

Moreover, we have demonstrated that other structural parameters impact the control energy in brain networks. Specifically, our result from studied brain networks indicates that a higher number of paths between brain regions results in less energy needed for controllability.

## Data Availability

The IBMB data are available from http://ibmb.nbml.ir/ on reasonable request. The data used during this study are also available from the corresponding author, upon reasonable request.
